# Response of Photosynthesis to High Growth Temperature Was Not Related to Leaf Anatomy Plasticity in Rice (*Oryza sativa* L.)

**DOI:** 10.3389/fpls.2020.00026

**Published:** 2020-02-07

**Authors:** Desheng Yang, Shaobing Peng, Fei Wang

**Affiliations:** ^1^MARA Key Laboratory of Crop Ecophysiology and Farming System in the Middle Reaches of the Yangtze River, College of Plant Science and Technology, Huazhong Agricultural University, Wuhan, China; ^2^National Key Laboratory of Crop Genetic Improvement, Huazhong Agricultural University, Wuhan, China; ^3^Hubei Collaborative Innovation Center for Grain Industry, Yangtze University, Jingzhou, China

**Keywords:** rice, photosynthesis, leaf anatomy, stomata, vein

## Abstract

Photosynthesis is highly sensitive to high temperature stress, and with the rising global temperature, it is meaningful to investigate the response of photosynthesis to growth temperature and its relationship with leaf anatomy plasticity. We planted 21 cultivars including eight *indica* cultivars, eight *japonica* cultivars, and five *javanica* cultivars in pot experiments under high growth temperature (HT, 38/28°C, day/night) and control treatment (CK, 30/28°C, day/night). Photosynthetic rate (*A*), stomatal conductance (*g_s_*), transpiration rate (*E*), stomatal density (SD), vein density (VD), minor vein area (SVA), and major vein area (LVA) were measured after 30 treatment days. Results showed HT significantly increased *A*, *g_s_*, and *E*, while significantly decreased SD and LVA. There was no significant difference in *A* among the three subspecies both under CK and HT, while the *javanica* subspecies had higher *g_s_*, *E*, SVA, and LVA under HT, and the *indica* cultivars had higher VD and SD both under CK and HT. The *javanica* subspecies had higher relative value (HT/CK) of *A*, *g_s_*, and *E*, while difference was not observed in the relative value of SD, VD, and LVA among the three subspecies. The relative value of *A* was positively related to that of *g_s_*, while the latter was not correlated with the relative value of SD, VD, SVA, and LVA. Overall, the results suggested the increase of *A* and *g_s_* at HT was not attributed to leaf anatomy plasticity in respect of stomata and vein under HT.

## Highlights

1. High growth temperature (HT) significantly affected rice leaf photosynthetic rate (*A*), stomatal conductance (*g_s_*), transpiration rate (*E*), stomatal density (SD), and major vein area (LVA).
2. The *javanica* subspecies had higher *g_s_*, *E*, and LVA under HT, and possessed higher heat resistance than *indica* and *japonica* subspecies.
3. Across different cultivars, the response of *A* and *g_s_* to HT were not related to leaf anatomy plasticity such as stomatal and vein anatomy.

## Introduction

Rice is a stable food for more than half of the global population (Khush, 2013). China is the top rice producer in the world, accounting for almost 30% of the global rice production (Tang et al., 2014). However, after a remarkable 86% increase in cereal production from 1980 to 2005, recent crop yield growth in China has been slow (Huang and Rozelle, 2015). Further improvement of crop yield potential through crop improvement is the best way to increase the grain yield (Peng, 2014). Yield potential of grain crop is defined as the grain yield obtained under optimum conditions without pests, diseases, weeds, and other stresses (Evans and Fischer, 1999). It is determined by the following factors, i.e. the total incident solar radiation on the land throughout the growing season, the light interception efficiency of plant canopy, the photosynthetic conversion efficiency of leaves and the harvest index (Monteith, 1977). Over the past few decades, the yield potential has been successfully achieved through increasing the light interception efficiency and harvest index, there is limited scope for further improvement when they reach 0.9 and 0.6, respectively (Beadle and Long, 1985; Hay, 1995). Therefore, it is more effective to increase crop yield by increasing photosynthetic capacity, which is known as the new ‘green revolution' (Zhu et al., 2010; Long et al., 2015).

Global warming represents a continual challenge for agricultural production and food security. For example, each 1°C increase in growing season temperature can result in up to 17% decrease in corn and soybean yield (Lobell and Asner, 2003), and a 10% decrease in rice yield (Peng et al., 2004). The mean surface air temperature has increased globally by ∼1°C in the last 100 years and will further increase by 1 to 4°C in this century (Zhao et al., 2017). Nevertheless, photosynthesis is highly sensitive to high temperature stress and is often inhibited before other cell functions are impaired (Berry and Bjorkman, 1980). Photosynthesis usually peaks at ~30°C in rice plants, and CO_2_ assimilation may decrease significantly after suffering a heat stress (Yamori et al., 2011; Xiong et al., 2017). Mathur et al. (2014) concluded that high temperature stress mainly inhibited various redox and metabolic reactions taking place in Photosystem II, Photosystem I, Cytochrome complex, and Rubisco, eventually resulting in a decrease in photosynthetic rate (*A*). Huang et al. (2017) indicated that Rubisco activity, regeneration capacity of RuBP, rate of electron transport, and CO_2_ diffusion capacity are sensitive to temperature and negatively impacted by high temperature stress.

Under high temperature conditions, plants exhibit short-term avoidance or acclimation mechanisms such as transpirational cooling, stomatal closure, and so on (Mathur et al., 2014). This means a close relationship exist between CO_2_ delivery and water transportation in a leaf. Stomata, through which CO_2_ and water vapor diffuse into and out of the leaf, are involved in the regulation and control of photosynthesis and transpiration responses (Farquhar and Sharkey, 1982; Jones, 1998). Stomatal density (SD) and size are leaf anatomical traits contributing to build the leaf *g_s_* to gas diffusion (Franks et al., 2010). Under high temperature, stomata closure is another reason for impaired photosynthesis that affects the intercellular CO_2_ (Hasanuzzaman et al., 2013). The leaf venation system as water transport channel in vascular plants plays an important role in maintaining adequate *E* (Sack and Holbrook, 2006; Tholen et al., 2012). An increased VD may facilitate a higher photosynthetic capacity by allowing for more efficient photosynthate export from mesophyll cells (Amiard et al., 2005).

Rice is cultivated under a wide range of climatic conditions. *Indica* rice and *japonica* rice are two subspecies with different genotypic background that evolved from different temperature environment. *Indica* rice has stronger heat tolerance and is more suitable for high temperature environment than *japonica* rice (Weng and Chen, 1987). *Javanica* rice (tropical *japonica*) mainly distributes in tropical mountains of Indonesia, Malay Peninsula, the Philippines, e.g. which has strong resistance to abiotic stresses (Xiao and Yuan, 2009). Zhao et al. (2013) proposed that maintaining a higher level of photosynthesis and osmoregulation substance was the physiological basis for heat tolerance in *javanica* rice. Leaves have evolved in different environments showing great variation in morphology and anatomy, and investigating relationships between leaf anatomy and photosynthetic features can lead to the identification of structural features for enhancing crop productivity and improve our understanding of plant evolution and adaptation (Evans, 1998).

At present, there have been many studies about the effect of temperature treatment on photosynthesis, and the relationship between leaf anatomies and photosynthesis. However, the difference in response of *A* to HT among different subspecies (*indica*, *japonica*, and *javanica* rice) is still obscure. Exploring the natural genotypic variation in leaf anatomy plasticity in relation to photosynthesis at HT will facilitate rice genetic improvement especially under future climate conditions. Therefore, the objectives of the present study were to determine the contribution of *g_s_* to photosynthetic acclimation to HT for the three subspecies in rice, and examine its relationship with leaf plasticity.

## Materials and Methods

### Site Description and Growth Conditions

Pot experiments were conducted in controlled-growth chamber at the Huazhong Agricultural University, Wuhan city, Hubei province, China (114.37°E, 30.48°N) in 2017. In the chamber (Model GR48, Conviron, Controlled Environments Limited, Winnipeg, MB, Canada), the air temperature was set to 30/28°C (day/night, the control treatment) and 38/28°C (day/night, the high temperature treatment), with a relative humidity of 75%, photosynthetic photon flux density (PPFD) of 1,500 μmol m^−2^ s^−1^ and a light/dark regime of 12/12 h. Eleven-liter plastic pots were filled with 10.0 kg air-dried, pulverized, and well-mixed soil taken from the top 25 cm layer of a field located at the Experimental Station in the campus. The soil used in this study was a clay loam with a pH of 7.1, organic matter of 6.7g kg^−1^, Olsen-P of 6.27 mg kg^−1^, exchangeable K of 129 mg kg^−1^, and total N of 0.063%.

### Experimental Design and Crop Management

Twenty-one rice cultivars were used in present study, including eight *indica* cultivars: Shenglixian (SLX), Huangguaxian (HGX), Zhenzhuai (ZZA), Ezhong2 (EZ2), Guichao2 (GC2), Huanghuazhan (HHZ), Yliangyou900 (YLY900), and Yangliangyou6 (YLY6), eight *japonica* cultivars: Guihuaqiu (GHQ), Guihuahuang (GHH), Xudao2 (XD2), Yanjing2 (YJ2), Zhendao88 (ZD88), Huaidao5 (HD5), Huaidao9 (HD9), and Lianjing7 (LJ7), and five *javanica* cultivars: PEMBE (J-1), TREMBESE (J-2), ASE BOLONG KAMANDI (J-3), PADI SEGUTUK (J-4), and BULUH BAWU (J-5) (**Table S1**). Particularly, the eight *indica* cultivars and eight *japonica* cultivars we used were historical cultivars from 1940s to present in China. Pre-germinated seeds were sown in a nursery plates on 27 August and transplanted to pots on 11 September with a density of three hills per pot and two seedlings per hill. Each cultivar was planted in six pots with three replicates. Equal pots were transferred to two growth chambers with different temperature treatments on 20 September. Other managements: 0.8 and 0.6 g N as urea per pot were applied at 1 d before transplanting and 7 d after transplanting, respectively; 1.5 g P as monocalcium phosphate and 1.5 g K as potassium chloride per pot were applied at 1d before transplanting. Three to 5 cm of standing water was kept in the pots throughout the experiment. Weeds were removed manually. Pests and diseases were controlled by chemicals two to three times. The measurements were conducted at the maximal tillering stage, on 20 October.

### Measurement of Leaf Gas Exchange

Gas exchange measurements were conducted at 30 days after treatment (or 53 days after sowing at tillering stage) from 09:00 h to 16:00 h on the newest fully expanded leaves using a portable photosynthesis system (LI-6400XT; LI-COR Inc., Lincoln, NE, USA) with a 6400-40 leaf chamber. In the leaf chamber, the PPFD was maintained at 1,500 μmol m^−2^ s^−1^, the leaf-to-air vapor pressure deficit (VPD) was 1.5 to 2.0 kPa, and the CO_2_ concentration was adjusted to 400 μmol mol^−1^ using a CO_2_ mixer. The block temperature during the measurement was set to the same as the growth condition temperature. After equilibration to a steady state, the gas exchange parameters including net photosynthesis rate (*A*), *g_s_*, and *E* were recorded.

### Measurement of Leaf Anatomy

After measuring the photosynthetic parameters, the middle part of the corresponding leaf blade was sampled. Half of it was stored in a distilled water to measure the leaf SD and VD. The other half of the leaf was cut into slices broadwise for determination of leaf vascular bundle anatomies, including SVA and major vein area (LVA). Specifically, the inverted fluorescence microscope (U-TVO.5XC; Olympus, Tokyo, Japan) was used to observe and photograph the leaf vein, stomata, and vein anatomies. The number of leaf stomata and vein, as well as the length and width of major vein and minor vein in each photograph were manually measured using ImageJ (Wayne Rasband/NIH, Bethesda, MD, USA). The leaf stomata density and VD were calculated by the formula: no./actual area, and the SVA and major vein area were calculated by the formula: *π* * ((length + width)/4)^2^.

### Statistical Analysis

Two-way analysis of variance (ANOVA) was used to assess the effects of high temperature and subspecies on each parameter using Statistix 9 software (Analytical Software, Tallahassee, Florida, USA). Linear regression analysis was performed to test the correlations between *g_s_* and *A*, SD and *g_s_*, and VD and vein area using SigmaPlot 12.5 (Systat Software Inc., California, USA).

## Results

### Photosynthetic Rate, Stomatal Conductance, Transpiration Rate, and Intrinsic Water Use Efficiency (WUEi)

**Figure 1:**
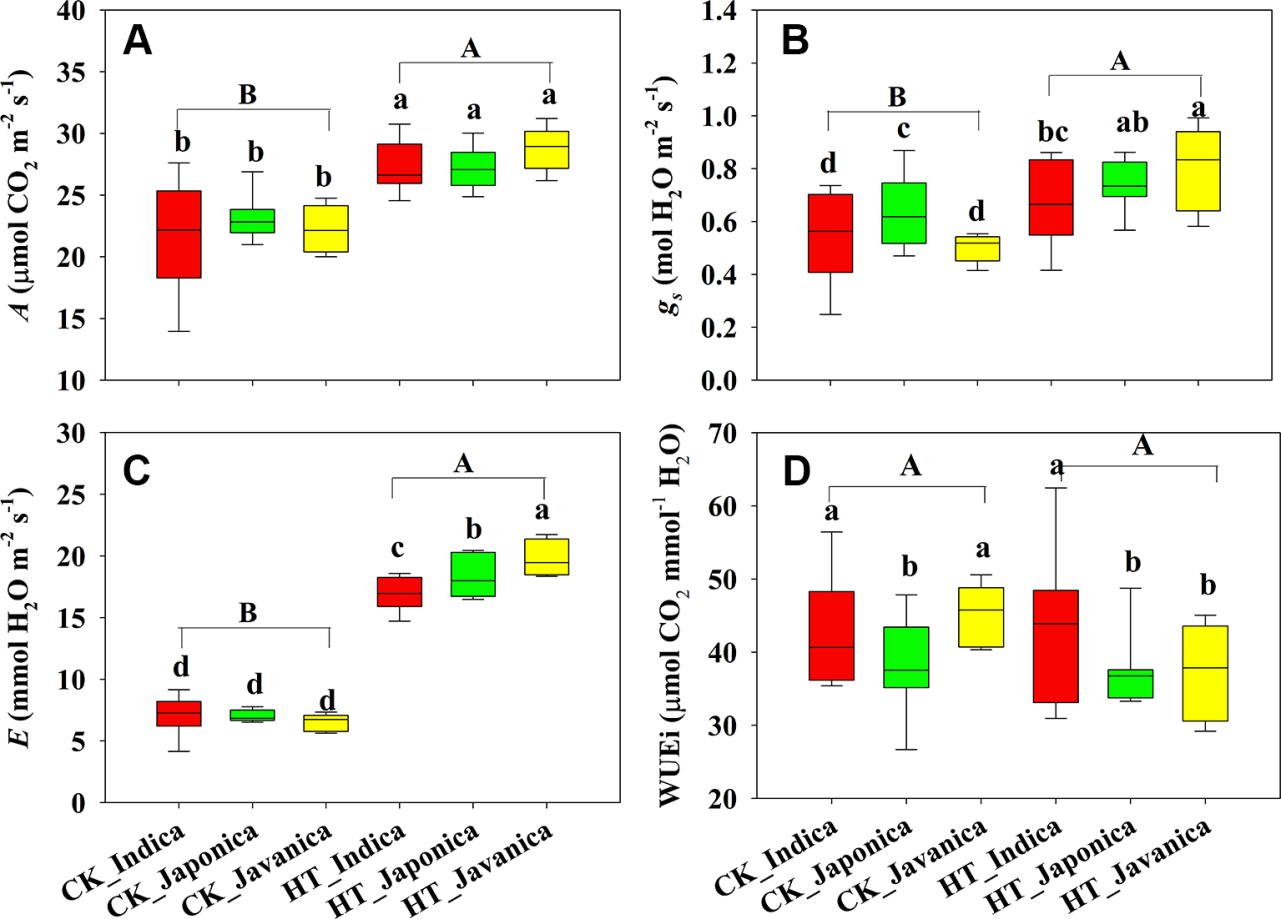
Light saturated photosynthetic rate (*A*, **A**), stomatal conductance (*g_s_*, **B**), transpiration rate (*E*, **C**), and intrinsic water use efficiency (WUEi, **D**) for the three rice (*Oryza sativa* L.) subspecies at high growth temperature (HT) and control (CK) treatments. Different lowercase and uppercase letters indicate significant differences between subspecies means and temperature treatments, respectively (p < 0.05). The outer box edges represent the 25th and 75th percentiles, the error bars are the 5th and 95th percentiles, and the line within the box represents the mean values. For each cultivar, three flag leaves were measured (one leaf per plant), and the three values were averaged as one biological replicate. n (the biological replicates) was 8, 8, and 5 for the indica, japonica, and javanica cultivars, respectively.

Temperature (T) significantly affected the *A*, but subspecies (S) had no significant effect on *A* (**Figure 1A**). Compared with the control treatment (CK), HT increased *A* by 23% averagely across all cultivars. There were no significant differences in A among the three subspecies both at high temperature and CK treatments. Stomatal conductance (*g_s_*) and *E* were significantly affected by temperature and subspecies (**Figures 1B, C**). HT increased *g_s_* and *E* by a mean value of 35% and 162%, respectively. *Japonica* cultivars had significantly higher *g_s_* than *indica* and *javanica* cultivars at CK, however, *javanica* cultivars had significantly higher *g_s_* than *indica* cultivars at HT treatment (**Figure 1B**). There was no significant difference in *E* among the three subspecies at CK, while *E* of *javanica* cultivars was significantly higher than that of *japonica* cultivars and *indica* cultivars had the lowest value (**Figure 1C**). At CK, *indica* and *javanica* cultivars had the similar WUEi higher than *japonica* cultivars, while WUEi of *indica* cultivars was significantly higher than that of *japonica* and *javanica* cultivars at HT (**Figure 1D**).

**Figure 2:**
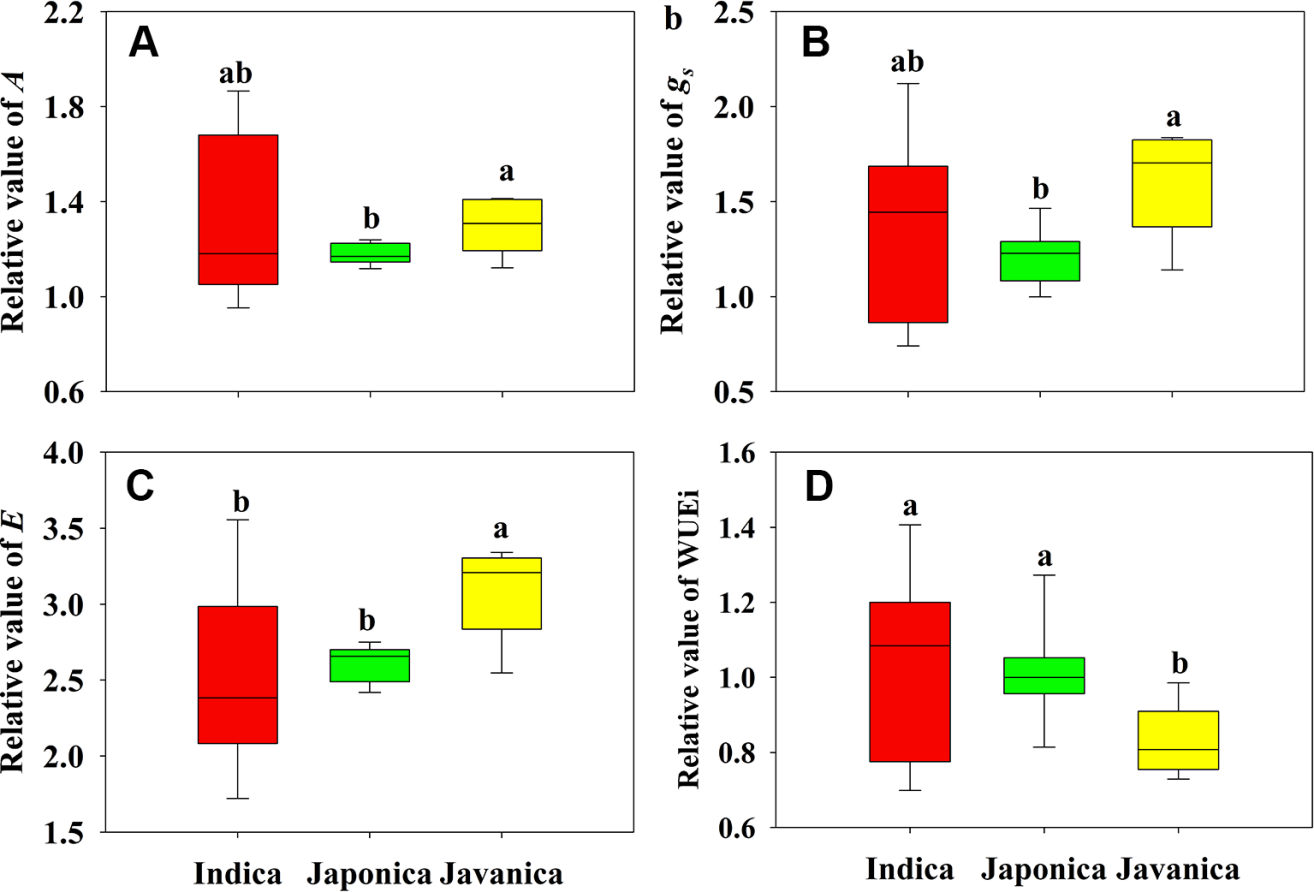
Relative value (HT/CK) of light saturated photosynthetic rate (*A*, **A**), stomatal conductance (*g_s_*, **B**), and transpiration rate (*E*, **C**), and intrinsic water use efficiency (WUEi, **D**) for the three rice (*Oryza sativa* L.) subspecies. Different lowercase letters indicate significant differences between subspecies means (p < 0.05). The outer box edges represent the 25th and 75th percentiles, the error bars are the 5th and 95th percentiles, and the line within the box represents the mean values. For each cultivar, three flag leaves were measured (one leaf per plant), and the three values were averaged as one biological replicate. n (the biological replicates) was 8, 8, and 5 for the indica, japonica, and javanica cultivars, respectively.

Different response of *A* to HT was observed among *indica*, *japonica*, and *javanica* subspecies (**Figure 2A**). Compared to that at CK, the increment of *A* of *indica*, *japonica*, and *javanica* subspecies at HT were 23%, 18% and 29% on average, respectively. The response of *A* to HT varied significantly among the *indica* cultivars with the relative value of *A* ranging from 0.95 to 1.87, while the responses of A to high temperature in *japonica* cultivars were consistent. Similar results in the response to HT between the three subspecies were observed for *g_s_* and *E* (**Figures 2B, C**). For WUEi, only *javanica* cultivars showed a reduction in WUEi under HT in comparison with CK (**Figure 2D**).

### Stomatal Density, Vein Density, and Vein Area

**Figure 3:**
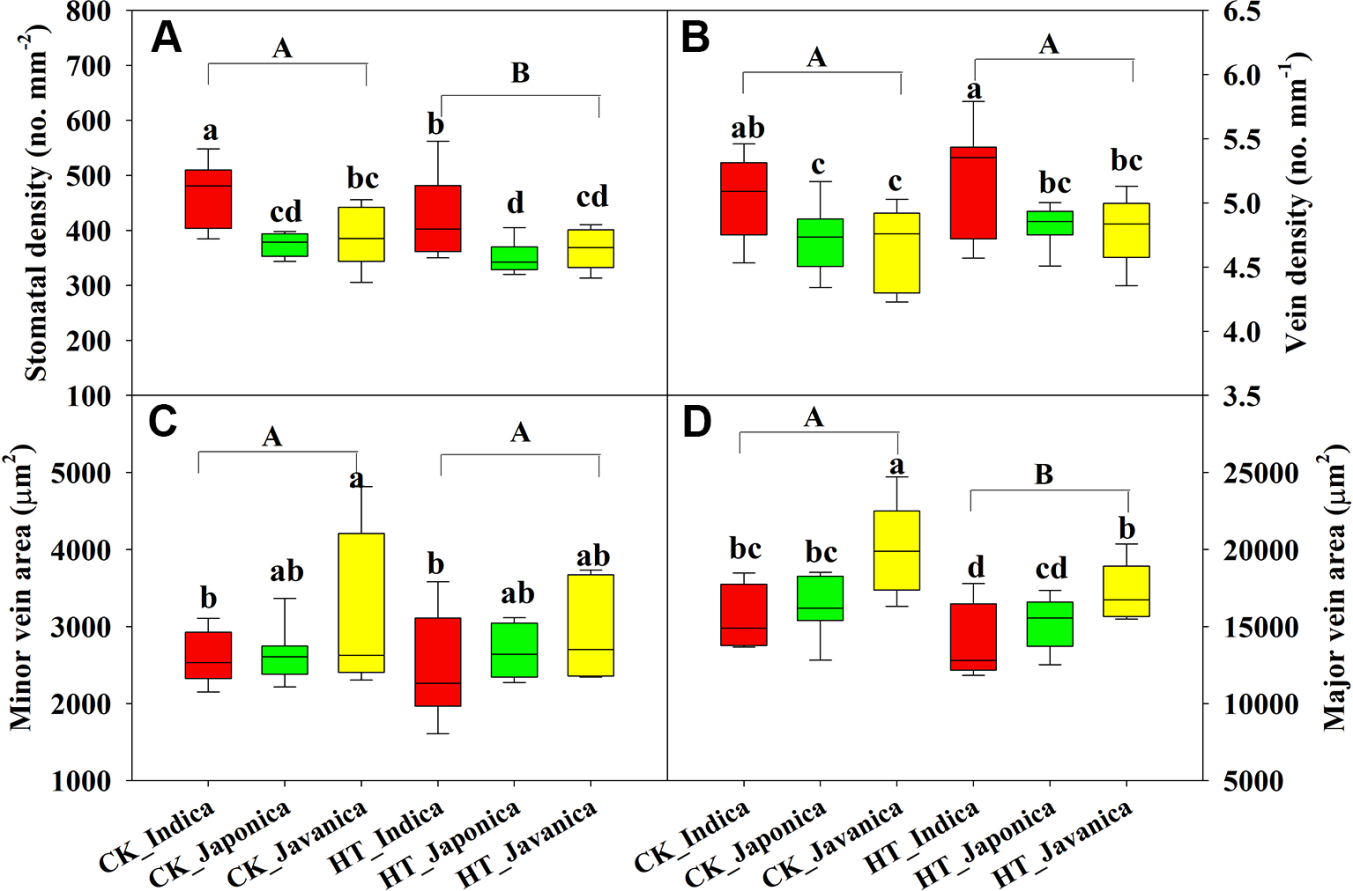
Stomatal density **(A)**, vein density **(B)**, and vein area of minor **(C)**, and major **(D)** veins for the three rice (*Oryza sativa* L.) subspecies at high growth temperature (HT) and control (CK) treatments. Different lowercase and uppercase letters indicate significant differences between subspecies means and temperature treatments, respectively (p < 0.05). The outer box edges represent the 25th and 75th percentiles, the error bars are the 5th and 95th percentiles, and the line within the box represents the mean values. For each cultivar, three flag leaves were measured (one leaf per plant), and the three values were averaged as one biological replicate. n (the biological replicates) was 8, 8, and 5 for the indica, japonica, and javanica cultivars, respectively.

Temperature significantly affected SD and major vein area (LVA), while no differences were observed in VD and SVA between HT and CK (**Figure 3**). Compared to CK, HT decreased SD and LVA by a mean value of 7% and 8.9%, respectively (**Figures 3A, D**). At both CK and HT, *indica* cultivars had significantly higher SD than *japonica* and *javanica* cultivars on average, moreover, the genotypic variation was also larger within *indica* cultivars (**Figure 3A**). Similar results were observed for the VD (**Figure 3B**). There were no significant differences in SD and VD between the *japonica* and *javanica* cultivars (**Figures 3A, B**). *Javanica* cultivars had the largest vein area, which were significantly higher than that of *indica* cultivars in minor vein (**Figure 3C**) and that of *indica* and *japonica* cultivars in major vein (**Figure 3D**).

**Figure 4:**
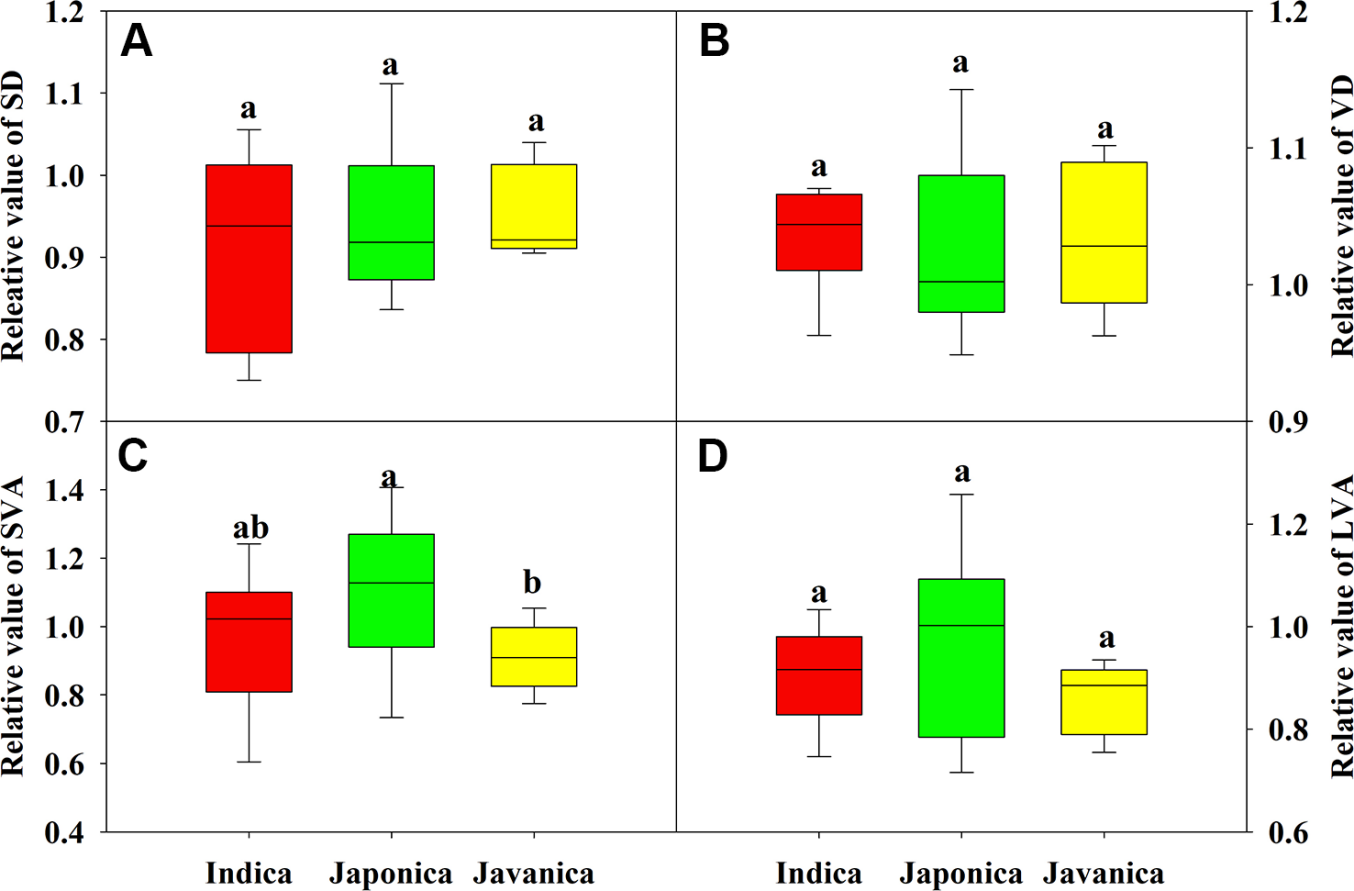
Relative value (HT/CK) of stomatal density (SD, **A**), vein density (VD, **B**), and vein area of minor **(C)** and major **(D)** veins for the three rice (*Oryza sativa* L.) subspecies. Different lowercase letters indicate significant differences between subspecies means (p < 0.05). The outer box edges represent the 25th and 75th percentiles, the error bars are the 5th and 95th percentiles, and the line within the box represents the mean values. For each cultivar, three flag leaves were measured (one leaf per plant), and the three values were averaged as one biological replicate. n (the biological replicates) was 8, 8, and 5 for the indica, japonica, and javanica cultivars, respectively.

There were no significant differences in the response of SD, VD, and major vein area to HT (**Figures 4A, B, D**). *Japonica* cultivars had higher relative value of SVA than *javanica* cultivars (**Figure 4C**). Large variations were observed for all the leaf anatomy parameters within each subspecies, for example the relative value of SD ranged from 0.75 to 1.03 for the *indica* cultivars (**Figure 4**).

### Correlations Between Vein Density and Vein Area, *g_s_* and *A*, Stomatal Density, and *G_s_*

**Figure 5:**
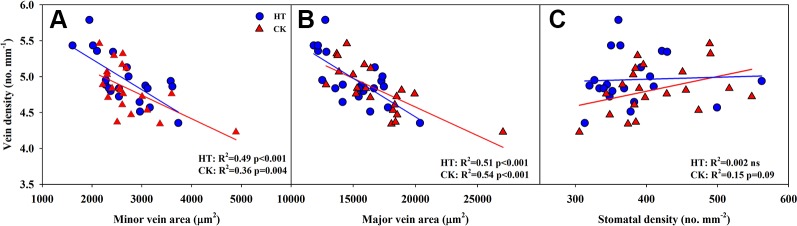
Correlations between vein density (VD) and vein area of minor vein (SVA, **A**) and major vein (LVA, **B**), and between VD and stomatal density (SD, **C**) at high growth temperature (HT) and control (CK) treatments for the three rice (*Oryza sativa* L.) subspecies.

**Figure 6:**
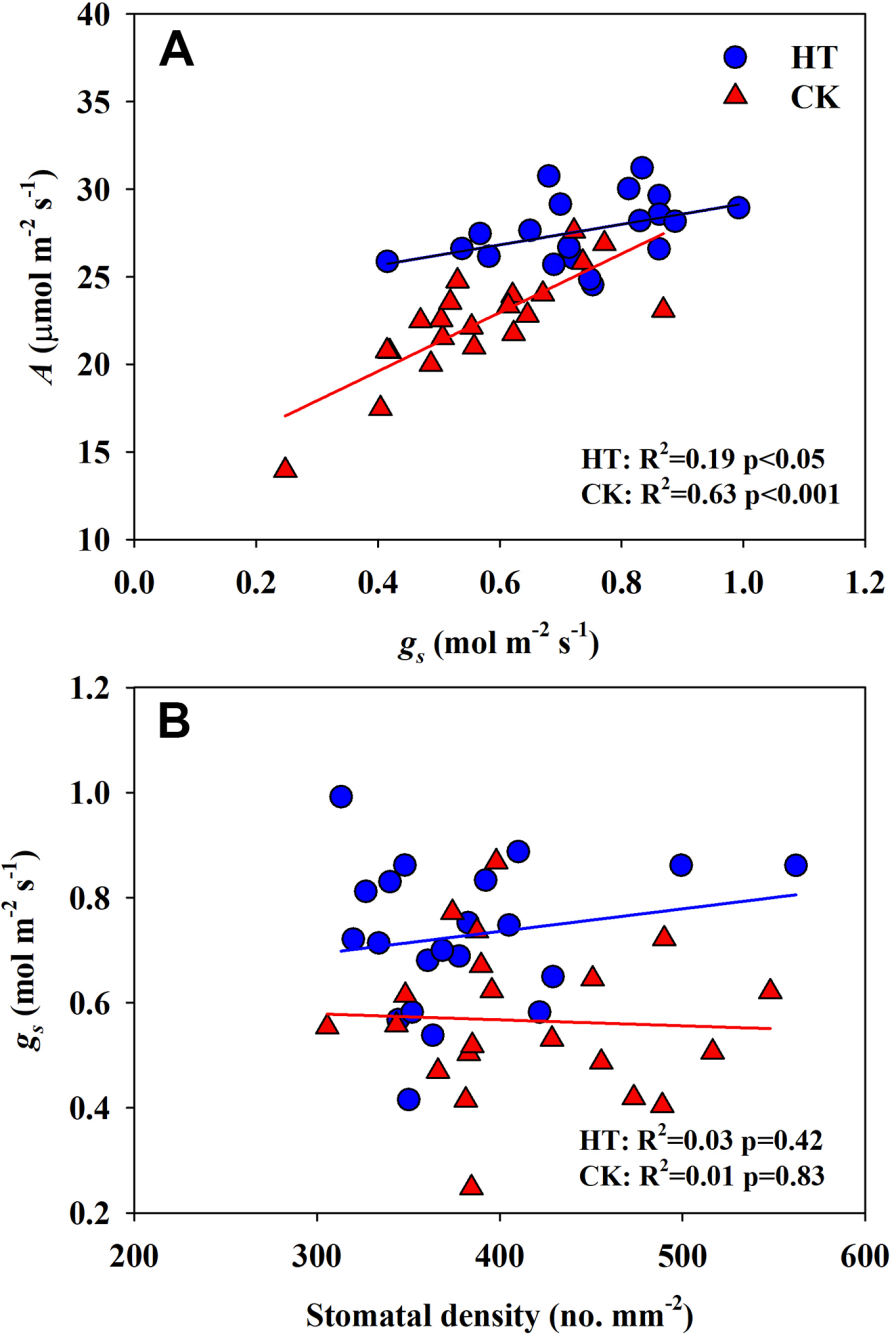
Correlations between light saturated photosynthetic rate **(A)** and stomatal conductance (*g_s_*), and between stomatal conductance (*g_s_*) **(B)** and stomatal density (SD) at high growth temperature (HT) and control (CK) treatments for the three rice (*Oryza sativa* L.) subspecies.

**Figure 7:**
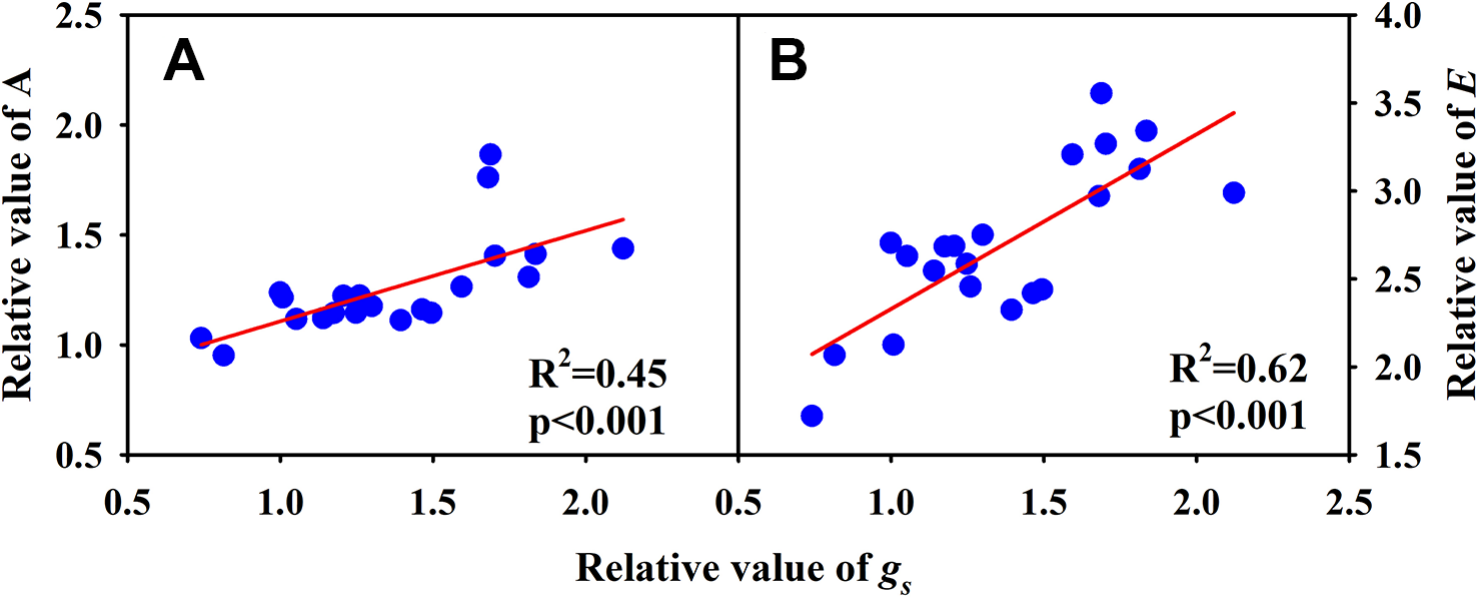
Correlations between the relative value of light saturated photosynthetic rate **(A)** and the relative value of stomatal conductance (*g_s_*), and between the relative value of transpiration rate **(B)** and conductance (*g_s_*) and the relative value of *g_s_* for the three rice (*Oryza sativa* L.) subspecies.

Significantly negative correlations between VD and SVA, as well as between VD and major vein area were observed both under CK and HT (**Figures 5A, B**). Stomatal density significantly correlated with VD at CK (*R*^2^ = 0.15), but not at HT treatment (**Figure 5C**). *A* was positively related to *g_s_* both under CK and HT (**Figure 6A**), but the correlation coefficient under CK was much larger than that under HT (*R*^2^ = 0.63 and 0.19), which was due to the integral higher *g_s_* under HT. Nevertheless, *g_s_* was not related to SD both under CK and HT (**Figure 6B**). The responses of *A* and *E* to HT were significantly correlated to the response of *g_s_* to HT (*R*^2^ = 0.45 and 0.62, **Figure 7**).

## Discussion

### Photosynthetic Acclimation to High Growth Temperature

In present study, HT significantly increased *A* of all cultivars except two *indica* cultivars, EZ2 and GC2 (**Figure 1** and **Figure S1**). Among the three subspecies, *javanica* (the tropical *japonica*) is closer to *japonica* at the genomic level (Wang et al., 2018). This was also manifested by the similar leaf anatomy of the two subspecies (**Figure 3**). However, *javanica* subspecies possessed higher heat resistance than *indica* and *japonica* subspecies in respect of the photosynthetic physiology, for example the response of *A*, *g_s_*, and *E* to high temperature (**Figure 2**). This is possible because *javanica* cultivars mainly distributes in tropical mountains with strong resistance to abiotic stresses (Xiao and Yuan, 2009). *Javanica* cultivars could maintain a higher level of photosynthesis and osmoregulation substance under HT (Zhao et al., 2013).

The thermal response curve of *A* usually peaks between 25 and 30°C in C_3_ photosynthetic species (Sage and Kubien, 2007; Yamori et al., 2014; Xiong et al., 2017), although some C_3_ species can maintain high *A* at temperatures as high as 45°C (e.g. Lawson et al., 2014). Two main biochemical hypotheses have been put forward to explain why *A* decreases above the optimum temperature: Rubisco activase heat lability and electron transport declines (Dusenge et al., 2019). It is notable that tissues that develop after a shift in temperature often show greater acclimation to that new temperature than those that developed prior to the change in temperature (i.e. “long-term” > “short-term”; e.g. Campbell et al., 2007). Many studies have shown that the response of *A* to temperature depends on the temperature experienced by the plant over longer time periods, a response termed temperature acclimation (Berry and Bjorkman, 1980; Hikosaka et al., 2006; Way and Yamori, 2014; Yamori et al., 2014). For example, Way and Yamori (2014) found that about half of the 103 species in their database had increased *A* in response to warming. Within C_3_ species, annual herbaceous plants (*A* of high temperature/low temperature, 1.31 ± 0.07) show greater *A* temperature response than evergreen woody plants (0.99 ± 0.03), deciduous woody plants (1.19 ± 0.12), and perennial herbaceous plants (1.03 ± 0.07, Yamori et al., 2014). In present study, rice plants were treated at HT for one month, and *A* at high temperature treatment was higher than that at control treatment by 23.7% on average.

### Physiological Mechanisms Underlying the Temperature Acclimation

The physiological mechanisms underlying photosynthetic acclimation of *A* to warmer temperatures have been extensively investigated (Sage and Kubien, 2007; Yamori et al., 2014). Proton leakiness of the thylakoid membrane has been frequently proposed as a problem at high temperatures, since it could lead to the impairment of the coupling of ATP synthesis to electron transport. Increases in cyclic electron flow around PSI at high temperature can compensate for thylakoid leakiness, allowing ATP synthesis to continue (Bukhov et al., 2000; Yamori et al., 2014). In many plant species, the Rubisco activation state decreases at short-term high temperature due to the insufficient activity of Rubisco activase, however, in plants adapted to HT, a different isoform of Rubisco activase that confers heat stability can be produced by some species, including spinach, cotton, and wheat (Yamori et al., 2014). Moreover, expression of heat-shock proteins (HSPs)/chaperones at high temperature contributes to the temperature acclimation through their effects in protein folding and assembly, stabilization of proteins and membranes, and for cellular homeostasis at high temperature (Barua et al., 2003).

Under high temperature, transpirational cooling is also an important acclimation mechanism in plants (Crawford et al., 2012; Mathur et al., 2014). Heat stress may be somewhat mitigated if transpiration-mediated cooling can be maintained, for example, rice can remain productive in air temperatures of 40°C if humidity remains low (Jagadish et al., 2014). In Blueberry, the thermal tolerant cultivars could improve their heat dispersing efficiency through regulating stomatal traits (Hao et al., 2019). In present study, the *E* of all cultivars was significantly increased under high temperature. The increase in *A* and *E* at HT was mainly related to the significantly increased *g_s_* (**Figures 6** and **7A**).

### Anatomical Mechanisms Underlying the Temperature Acclimation

Photosynthetic acclimation to long-term high temperature may be partly due to the structural changes in leaf tissues (Sage and Kubien, 2007; Yamori et al., 2014). In higher plants, water from the stem enters the petiole and moves through xylem in different vein orders, then exits into the bundle sheath and moves through mesophyll tissue before evaporating into the intercellular airspace and diffusing through stomata (Xiong et al., 2016). Stomatal density, leaf vein systems include VD and vascular bundle features, were strongly correlated with the hydraulic conductivity and maximum *A* (Brodribb et al., 2007). In present study, there was no significant difference in the response of SD, VD, and vein area to high temperature between the three subspecies (**Figure 4**), however, significant genotypic variation was found within each subspecies (**Figure S2**). High temperature significantly reduced SD and SVA (**Figures 3A, D**). This was consistent with previous studies in the model species *Arabidopsis thaliana* and Scots pine (*Pinus sylvestris* L.) (Luomala et al., 2005; Vile et al., 2012). Crawford et al. (2012) illustrated that increased stomatal spacing by lowering SD, may facilitate evaporative cooling in high temperature conditions, through increasing the inter−stomatal space available for vapor diffusion. However, some other studies found significant increase in SD at high temperature in soybean (Jumrani et al., 2017) and rice (Caine et al., 2018). Therefore, more studies are needed to examine the plasticity of other leaf structures in response to high temperature and its effects on photosynthetic physiology.

## Conclusion

The current study revealed rice leaf photosynthetic physiology exhibited high temperature acclimation, and the *javanica* subspecies possessed higher heat resistance than *indica* and *japonica* subspecies. Large genotypic variation in response of *A*, *g_s_*, *E*, and other leaf anatomies to HT as well as correlation analysis showed the response of *A* and *g_s_* among different cultivars to HT was not related to the plasticity of leaf anatomy (mainly the stomatal and vein structure).

## Data Availability Statement

All datasets generated for this study are included in the article/**Supplementary Material**.

## Author Contributions

FW conceived and designed the research. DY conducted the experiments and collected the data, DY and FW analyzed the data and wrote the paper. FW and SP commented and revised the paper.

## Funding

This work was supported by the Program of National Natural Science Foundation of China (No. 31501255), the National Key Research and Development Program of China (No. 2017YFD0301401-3).

## Conflict of Interest

The authors declare that the research was conducted in the absence of any commercial or financial relationships that could be construed as a potential conflict of interest.
