# Supplementary material for: Response of Photosynthesis to High Growth Temperature Was Not Related to Leaf Anatomy Plasticity in Rice (Oryza sativa L.)
Source: Front Plant Sci. 2020 Feb 7;11:26. doi: 10.3389/fpls.2020.00026 (PMC7018767; doi:10.3389/fpls.2020.00026)
Supplement: Supplementary file 1 [file DataSheet_1.docx]

Table S1 Information of the rice (*Oryza sativa* L.) cultivars. The information was obtained from the website of China Rice Data Center (http://www.ricedata.cn/variety/).

| **Abbreviation** | **Cultivar** | **Subspecies** | **Type** | **Country** | **Province** |
| --- | --- | --- | --- | --- | --- |
| SLX | Shenglixian | Indica | Inbred | China | Hunan |
| HGX | Huangguaxian | Indica | Inbred | China | Anhui |
| ZZA | Zhenzhuai | Indica | Inbred | China | Guangdong |
| EZ2 | Ezhong2 | Indica | Inbred | China | Hubei |
| GC2 | Guichao2 | Indica | Inbred | China | Guangdong |
| HHZ | Huanghuazhan | Indica | Inbred | China | Guangdong |
| YLY900 | Yliangyou900 | Indica | hybrid | China | Hunan |
| YLY6 | Yangliangyou6 | Indica | hybrid | China | Jiangsu |
| GHQ | Guihuaqiu | Japonica | Inbred | China | Anhui |
| GHH | Guihuahuang | Japonica | Inbred | China | Jiangsu |
| XD2 | Xudao2 | Japonica | Inbred | China | Jiangsu |
| YJ2 | Yanjing2 | Japonica | Inbred | China | Jiangsu |
| ZD88 | Zhendao88 | Japonica | Inbred | China | Jiangsu |
| HD5 | Huaidao5 | Japonica | Inbred | China | Jiangsu |
| HD9 | Huaidao9 | Japonica | Inbred | China | Jiangsu |
| LJ7 | Lianjing7 | Japonica | Inbred | China | Jiangsu |
| J-1 | Pembe | Javanica | - | - | - |
| J-2 | Trembese | Javanica | - | Indonesia | - |
| J-3 | ASE BOLONG KAMANDI | Javanica | - | Indonesia | - |
| J-4 | PADI SEGUTUK | Javanica | - | - | - |
| J-5 | BULUH BAWU | Javanica | - | - | - |

Table S2 Light saturated photosynthesis rate (*A*), stomatal conductance (*g_s_*), transpiration rate (*E*), stomatal density (SD), leaf vein density (VD), minor vein area (SVA) and major vein area (LVA) for the three subspecies of rice (*Oryza sativa* L.) at high growth temperature (HT) and the control (CK) treatments

| Treatment | Type | Cultivar | *A*  (μmol CO_2_ m^-2^ s^-1^) | *g_s_*  (mol H_2_O m^-2^ s^-1^) | *E*  (mmol H_2_O m^-2^ s^-1^) | SD  (no. mm^-2^) | VD  (no. mm^-1^) | SVA  (μm^2^) | LVA  (μm^2^) |
| --- | --- | --- | --- | --- | --- | --- | --- | --- | --- |
| CK | Indica | SLX | 14.0 f | 0.25 d | 4.2 d | 384 b | 5.1 ab | 2684 ab | 16403 ab |
|  |  | HGX | 17.5 e | 0.41 c | 6.2 c | 489 a | 5.43 a | 2148 b | 16015 ab |
|  |  | ZZA | 20.7 d | 0.42 c | 6.3 c | 473 ab | 4.53 b | 3107 a | 18201 a |
|  |  | EZ2 | 27.6 a | 0.72 a | 8.3 ab | 490 a | 5.3 a | 2618 ab | 13676 b |
|  |  | GC2 | 25.8 ab | 0.74 a | 9.2 a | 387 b | 5.3 a | 2441 b | 13750 b |
|  |  | HHZ | 22.8 cd | 0.65 ab | 8.0 b | 451 ab | 5.07 ab | 2299 b | 13857 b |
|  |  | YLY900 | 23.9 bc | 0.62 b | 7.8 b | 548 a | 4.73 ab | 3006 a | 18491 a |
|  |  | YLY6 | 21.5 cd | 0.51 c | 6.7 c | 517 a | 4.83 ab | 2402 b | 15313 ab |
|  | Japonica | GHQ | 21.8 cd | 0.62 bcd | 6.5 c | 396 a | 5.17 a | 2604 ab | 15897 ab |
|  |  | GHH | 22.6 bcd | 0.51 de | 6.8 bc | 383 a | 4.63 bc | 2607 ab | 18340 a |
|  |  | XD2 | 23.3 bc | 0.61 cde | 6.8 bc | 348 a | 4.47 bc | 2785 ab | 18532 a |
|  |  | YG2 | 22.5 bcd | 0.47 e | 6.6 c | 366 a | 4.87 ab | 2214 b | 12819 b |
|  |  | ZD88 | 23.1 bc | 0.87 a | 7.6 ab | 398 a | 4.7 bc | 2321 b | 16377 ab |
|  |  | HD5 | 26.9 a | 0.77 ab | 7.8 a | 374 a | 4.37 c | 3364 a | 18069 a |
|  |  | HD9 | 21.0 d | 0.56 cde | 6.8 bc | 344 a | 4.77 abc | 2624 ab | 15227 ab |
|  |  | LG7 | 24.0 b | 0.67 bc | 7.3 abc | 390 a | 4.83 ab | 2548 b | 16011 ab |
|  | Javanica | PEMBE | 23.5 a | 0.52 ab | 7.3 a | 385 ab | 4.37 bc | 2505 bc | 18397 bc |
|  |  | TREMBESE | 22.1 ab | 0.55 a | 6.7 ab | 306 b | 4.23 c | 4818 a | 24717 a |
|  |  | KAMANDI | 20.8 b | 0.41 b | 5.7 c | 381 ab | 5.00 a | 2303 c | 16321 c |
|  |  | SEGUTUK | 20.0 b | 0.49 ab | 5.9 bc | 456 a | 4.80 a | 2625 bc | 19878 b |
|  |  | BAWU | 24.8 a | 0.53 ab | 6.8 ab | 429 a | 4.77 ab | 3599 ab | 20268 b |
| HT | Indica | SLX | 25.9 cd | 0.41 f | 14.7 d | 350 d | 5.43 ab | **1608 d** | **11827 c** |
|  |  | HGX | 30.8 a | 0.68 bc | 18.3 ab | 361 cd | 5.77 a | 1946 cd | 12753 bc |
|  |  | ZZA | 29.6 ab | 0.86 a | 18.6 a | 499 ab | 4.57 c | 3162 a | 17792 a |
|  |  | EZ2 | 26.2 cd | 0.58 de | 17 abc | 422 cd | 5.33 ab | 2100 cd | 12177 c |
|  |  | GC2 | 26.6 cd | 0.54 e | 15.8 cd | 363 cd | 5.4 ab | 2018 cd | 12166 c |
|  |  | HHZ | 27.6 bc | 0.65 cd | 16.9 bc | 429 bc | 5.37 ab | 2422 bc | 12844 bc |
|  |  | YLY900 | 26.6 cd | 0.86 a | 18.1 ab | 562 a | 4.93 bc | 3580 a | 17264 a |
|  |  | YLY6 | 24.5 d | 0.75 b | 16.3 c | 383 cd | 4.67 c | 2955 ab | 14148 b |
|  | Japonica | GHQ | 24.9 e | 0.75 abc | 16.8 b | 405 a | 5.0 a | 2734 abc | 17354 a |
|  |  | GHH | 26.1 de | 0.72 bc | 16.5 b | 320 b | 4.87 a | 3072 a | 15644 abc |
|  |  | XD2 | 26.7 cde | 0.71 bc | 18.1 b | 334 b | 4.83 ab | 2541 abc | 13564 cd |
|  |  | YG2 | 27.5 bcd | 0.57 d | 17.8 b | 345 ab | 4.87 a | 2269 c | 14165 bcd |
|  |  | ZD88 | 28.6 ab | 0.86 a | 20.4 a | 348 ab | 4.73 ab | 2540 abc | 15445 abc |
|  |  | HD5 | 30.0 a | 0.81 abc | 20.4 a | 327 b | 4.93 a | 2278 bc | 12514 d |
|  |  | HD9 | 25.7 de | 0.69 cd | 16.7 b | 378 ab | 4.50 b | 2963 ab | 16366 ab |
|  |  | LG7 | 28.2 abc | 0.83 ab | 20.0 a | 340 ab | 4.83 ab | 3117 a | 16670 ab |
|  | Javanica | PEMBE | 26.2 c | 0.58 d | 18.6 c | 352 ab | 4.8 ab | 2374 c | 15826 a |
|  |  | TREMBESE | 28.9 b | 0.99 a | 21 ab | 313 b | 4.37 b | 3733 a | 20370 a |
|  |  | KAMANDI | 29.1 b | 0.70 cd | 18.4 c | 369 ab | 4.83 ab | 2341 c | 15480 a |
|  |  | SEGUTUK | 28.2 b | 0.89 ab | 19.5 bc | 410 a | 4.87 a | 3615 ab | 17470 a |
|  |  | BAWU | 31.2 a | 0.83 bc | 21.7 a | 392 a | 5.13 a | 2700 bc | 16735 a |

Within a column for each subspecies, means followed by different letters are significantly different at 0.05 probability level according to Least Significant Difference (LSD).

Fig.S1 The relative value of light saturated photosynthetic rate (*A*), stomatal conductance (*g_s_*), and transpiration rate (*E*) for the three subspecies.

Fig.S2 The relative value of stomatal density (SD), vein density (VD), minor vein area (SVA), major vein area (LVA) for the three subspecies.

Fig.S3 Correlation between the relative value of *g_s_* with the relative value of SD, VD, SVA, and LVA
